# Adaptive NK cells undergo a dynamic modulation in response to human cytomegalovirus and recruit T cells in in vitro migration assays

**DOI:** 10.1038/s41409-022-01603-y

**Published:** 2022-02-17

**Authors:** Débora Basílio-Queirós, Letizia Venturini, Susanne Luther-Wolf, Elke Dammann, Arnold Ganser, Michael Stadler, Christine S. Falk, Eva M. Weissinger

**Affiliations:** 1 Department of Hematology, Hemostasis, Oncology and Stem Cell Transplantation, Transplantation Biology Laboratory, Hannover Medical School, Hanover, Germany; 2 German Center for Infection Research (DZIF), Site Hannover-Braunschweig, Brunswick, Germany; 3 Hannover Medical School, Institute of Transplant Immunology, Hanover, Germany

**Keywords:** Translational research, Preclinical research, Risk factors

## Abstract

Human cytomegalovirus (HCMV) reactivation remains a relevant complication after hematopoietic stem cell transplantation (HSCT) despite the great progress made in prophylaxis and treatment. Adaptive Natural Killer (NK) cells undergo a persistent reconfiguration in response to HCMV reactivation however, the exact role of adaptive NK cells in HCMV surveillance is currently unknown. We studied the relationship between HCMV reactivation and adaptive NK cells in 70 patients monitored weekly until day +100 after HSCT. Absolute cell counts of adaptive NK cells increased significantly after resolution of HCMV-reactivation compared to patients without reactivation. Patients with HCMV-reactivation had an early reconstitution of adaptive NK cells (“Responders”) and had mainly a single reactivation (75% Responders vs 48% Non-Responders). Adaptive NK cells eliminated HCMV-infected human foreskin fibroblasts (HFF) in vitro and recruited T cells in an in vitro transwell migration assay. An extensive cytokine/chemokine panel demonstrated strongly increased secretion of CXCL10/IP-10, IFN-α, IL-1α, IL-1β, IL-5, IL-7 and CCL4. Thus, adaptive NK cells may control viral spread and T cell expansion and survival during HCMV-reactivation. Taken together, we have demonstrated the potential of adaptive NK cells in the control of HCMV reactivation both by direct cytotoxicity and by recruitment of other immune cells.

## Abstract (web summary)

Human cytomegalovirus (HCMV) is a β-herpes virus that causes life-long infection as a latent virus in its host. Its prevalence depends on socioeconomic geographical factors and can affect 50–90% of the population depending on these factors. HCMV infection is usually asymptomatic in healthy individuals. However, in patients lacking proper immune responses, such as following hematopoietic stem cell transplant (HSCT), HCMV can reactivate and increase the mortality and morbidity rates in these patients. We set to investigate the role of a population of innate cells, the adaptive Natural Killer (NK) cells, in the response to HCMV reactivation after HSCT. Our findings revealed that adaptive NK cells are modulated in response to HCMV reactivation after HSCT. Furthermore, in addition to their ability to eliminate HCMV-infected target cells after in vitro expansion, we have also shown that adaptive NK cells recruit T cells in response to co-culture with HCMV-infected target cells and identified secreted factors possibly involved in this recruitment.

## Introduction

Allogeneic hematopoietic stem cell transplantation (HSCT) remains a curative treatment for malignant and even some non-malignant hematological disorders [1]. Despite the great progress made in the reduction of toxicities of conditioning regimens and improvements in immunosuppression, HSCT is still hampered by severe complications. Next to relapse and graft-versus-host-disease infections, especially viral reactivations, lead to a significant increase of morbidity and even mortality [2–5]. HCMV has a prevalence of 50–90% in the human population, depending on age, geographical and socioeconomic factors [6]. After primary infection HCMV establishes a life-long latent infection [7]. Reactivation of HCMV can occur in up to 70% of seropositive recipients (R+) even when transplanted from HCMV-seropositive donors (D+). In addition to the previously mentioned increases in morbidity and mortality rates, HCMV-reactivations (HCMV-R) also lead to a reduction of quality of life due to prolonged hospitalization [8, 9].

While the role of T cells in the control of HCMV-R is quite accepted and understood to date, Natural Killer (NK) cells may also play an important role in immune reconstitution and surveillance. NK cells are innate lymphocytes well described by their ability to kill virally infected or malignant cells. NK cells are the main lymphocytic population to reconstitute early after HSCT [10]. The crucial role of NK cells in viral infection control is associated not only to the early reconstitution of this cell population (when other major players of the immune response are still lacking) but also by their particular response to different viruses. For instance, different viruses will promote the expansion of different NK cell subpopulations and trigger different cytotoxic/regulatory approaches. These tailored responses have been described for several viral infections, included among others, HCMV, Hepatitis B virus, Epstein-Barr virus and Human Immunodeficiency virus. This has been discussed in more length by Zuo and Zhao [11]. Furthermore, a prospective study by a Drylewicz et al. suggests that the rapid reconstitution of CD4^+^ T cells and NK cells (rather than the cytotoxic T cell population), is associated with the absence of HCMV-R after HSCT [12].

More recently, a subpopulation of NK cells expressing NKG2C has been identified and is increased in HCMV-seropositive healthy individuals. In addition, NKG2C^+^ cells expand upon co-cultivation with HCMV-infected fibroblasts [13, 14]. This NK cell subpopulation preferentially expresses CD57 and shows downregulation of activating markers such as NKp30 and NKp46 compared to conventional NK cells [15, 16]. Furthermore, HCMV infection shapes the NK cell repertoire in humans. Murine studies demonstrated that adoptively transferred adaptive NK cells protected against MCMV infection/ reactivation [13, 17]. Thus, these NK cells may play an adaptive-like role and are called memory-like or adaptive NK cells. Adaptive NK cells are detected in healthy HCMV-seropositive individuals, but are quite heterogeneous which may be due to the peptide-specific recognition of HCMV strains [18]. It is still unknown how adaptive NK cells are able to specifically respond to HCMV but their potential role in the immune reconstitution after HSCT is of great interest [19]. In addition, adaptive NK cells exhibit characteristics belonging to both the innate and adaptive branches of the immune system. For this reason, their possible role in bridging these two sides is also of great clinical interest. To date, the interaction of the innate and the adaptive immune system are of great interest. Several publications have shown that both NK and T cells need to be expanding to protect against recurrent CMV reactivations. Here we show that adaptive NK cells secrete a number of cytokines involved in T cell expansion and using migration assays we show that T cells migrate toward only the stimulated adaptive NK cells.

In the present study, we analyzed the reconstitution, expansion and function of adaptive NK cells in the context of HCMV reactivation in HCMV-seropositive donors and/or recipients pairs after HSCT.

## Results

### Adaptive NK cells expand and persist during and after HCMV reactivation

Peripheral blood (PB) samples from 70 HCMV-seropositive patient/donor pairs were examined for differential reconstitution of adaptive NK cells weekly up to day +100 after HSCT. Clinical and demographic data of the patients are summarized in Table 1. HCMV-serostatus of recipient and donor, number of patients with HCMV-R and the median day of HCMV-R are summarized in Table 2.

**Table 1:** Clinical and demographic parameters of patients included in the cohort.

Age	Median 58 (range 19–73)
Disease	
Acute (AML, ALL, sAML, s/tAML)	46 (65.7%)
Chronic (MDS, MPS, CML, CLL, MM, MD/MPN)	14 (20%)
Lymphoma (NHL, HD)	6 (8.6%)
Non-malignant (AA, PNH)	4 (5.7%)
Conditioning	
Myeloablative	12 (17.1%)
RIC	58 (82.9%)
GvHD-Prophylaxis	
CSA/MMF	54 (77.1%)
CSA/MTX	12 (17.1%)
Other	4 (5.7%)
HLA-match	
Matched	56 (80%)
Mismatched	14 (20%)
Donor	
Related	20 (28.6%)
Unrelated	50 (71.4%)
Gender	
Female	29 (41.4%)
Male	41 (58.6%)

acute: *AML* acute myeloid leukemia, *ALL* acute lymphatic leukemia, *sAML* secondary AML, chronic: *MDS/MPS* myelodysplastic/proliferative syndrome, *CML* chronic myeloid leukemia, *CLL* chronic lymphatic leukemia, lymphoma: *NHL* non-Hodgkin lymphoma, *HD* Hodgkin disease, *MM* multiple myeloma; non-malignant: *(S)AA* severe or very severe aplastic anemia, *PNH* paroxysmal nocturnal hematouria, myeloablative conditioning: *RIC* reduced intensity conditioning, *PBSC* peripheral blood stem cells, *BM* bone marrow, *CB* cord blood, *ATG* anti-thymocyte globulin, *CSA* cyclosporine A, *MTX* methotrexate, *MMF* mycophenolate motefil, other: *MMF* tacrolimus (FK506), or different combinations of immunosuppressants.

**Table 2:** HCMV-serostatus and patient groups.

CMV serostatus	Number of patients
R^+^D^+^	42
R^+^D^−^	18
R^−^D^+^	10
CMV Reactivation	37 (52.9%)
CMV Reactivation day post-transplantation	37 (range 17–63)

*R*^*+*^*D*^*+*^ recipient and donor are anti-cytomegalovirus IgG-positive, *R*^*+*^*D*^*−*^ recipient is anti-cytomegalovirus IgG-positive, *D*^*−*^ donor is IgG and IgM-negative.

**Fig. 1:**
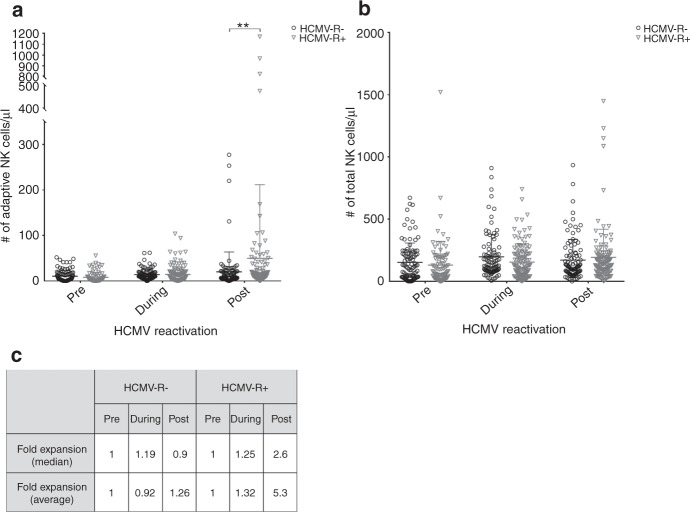
Absolute counts of adaptive NK cells in patients with or without HCMV reactivation. Cell aggregates were excluded by FSC-H vs FSC-A gating. SSC-A vs FSC-A gating discriminated between counting beads and lymphocytes. CD3, CD14 and 7AAD vs CD56 were used to discriminate NK cells from other lymphocytic populations. **a** Adaptive NK cells were selected as NKG2C-positive NK cells. Patients with HCMV reactivation (HCMV-R+) were analyzed in matched days post-HSCT with patients without HCMV reactivation (HCMV-R−). Absolute counts were calculated according to the formula: ${{{{{{{\rm{Adaptive}}}}}}}}\;{{{{{{{\rm{NK}}}}}}}}\;{{{{{{{\rm{cells}}}}}}}}/ \upmu {{{{{{{\rm{L}}}}}}}} = \frac{{{{{{{{{\rm{number}}}}}}}}\;{{{{{{{\rm{of}}}}}}}}\;{{{{{{{\rm{events}}}}}}}}\;{{{{{{{\rm{in}}}}}}}}\;{{{{{{{\rm{cell}}}}}}}}\;{{{{{{{\rm{population}}}}}}}}}}{{{{{{{{{\rm{number}}}}}}}}\;{{{{{{{\rm{of}}}}}}}}\;{{{{{{{\rm{events}}}}}}}}\;{{{{{{{\rm{in}}}}}}}}\;{{{{{{{\rm{absolute}}}}}}}}\;{{{{{{{\rm{count}}}}}}}}\;{{{{{{{\rm{bead}}}}}}}}\;{{{{{{{\rm{region}}}}}}}}}} \times \frac{{{{{{{{{\rm{number}}}}}}}}\;{{{{{{{\rm{of}}}}}}}}\;{{{{{{{\rm{known}}}}}}}}\;{{{{{{{\rm{beads}}}}}}}}\;{{{{{{{\rm{per}}}}}}}}\;{{{{{{{\rm{test}}}}}}}}}}{{{{{{{{{\rm{test}}}}}}}}\;{{{{{{{\rm{volume}}}}}}}}}}$. A diagnosis of HCMV reactivation was made when 5 or more pp65 antigen-positive cells per 4 × 10^5^ leukocytes were detected in the peripheral blood of a given patient. Resolution of HCMV reactivation was diagnosed after three consecutive negative pp65 antigen tests. **b** Absolute cell counts of unselected NK cells per µl of blood were calculated by the formula above. **c** The summary of the median and average-fold expansion of adaptive NK cells in patients without HCMV reactivation (HCMV-R−) and patients with HCMV-reactivation (HCMV-R+) is tabulated. Statistical analysis represents two-way ANOVA with Sidak’s multiple comparison test. Mean and standard deviation are shown. *n* = 70; **p < 0.01.

Patients with and without HCMV-R were analyzed as matched pairs according to days after HSCT. Figure 1a shows that although adaptive NK counts are low prior to HCMV-R, a dynamic modulation of absolute counts is seen during HCMV-R. An average 1.32 fold expansion of adaptive NK cells was observed in patients after HCMV-R compared to an average 0.92 fold increase in patients without HCMV-R (Fig. 1d). Remarkably, after resolution of the HCMV-R, these counts were significantly increased to an average 5.3 fold expansion (*p* < 0.01). Furthermore, after the resolution of HCMV-R, absolute counts of mature adaptive NK cells (CD57^+^ adaptive NK cells) were significantly increased in patients with HCMV-R compared to patients without HCMV-R (Fig. 1b). The comparison of absolute counts of total NK cells in patients with and without HCMV-R at the same time-points after HSCT is shown in Fig. 1c. The average-fold expansion of adaptive NK cells was independent of the increase of total NK cells in PB and thus not a consequence of the time after HSCT. Interestingly, overall NK cell counts were not influenced by the expansion of adaptive NK cells after HCMV-R.

### HCMV-R promotes a long-lasting increase in both HCMV-CTLs and adaptive NK cells

**Fig. 2:**
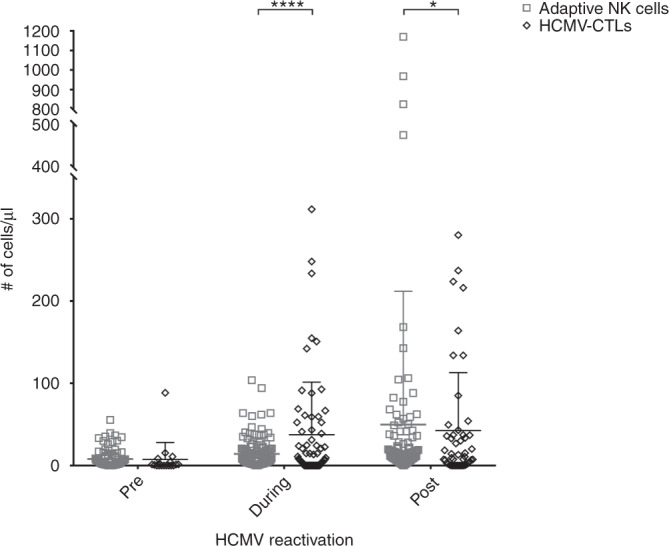
Absolute cell counts of adaptive NK cells and HCMV-CTLs. Patients were monitored for adaptive NK cell and HCMV-CTLs reconstitution after HSCT. Absolute cells counts are shown. Absolute counts of HCMV-CTLs were calculated according to the formula: ${{{{{\rm{HCMV}}}}}} \mbox{-} {{{{{\rm{CTLs}}}}}}/\upmu {{{{{\rm{L}}}}}} \!=\! \left({{{{{{\rm{\% }}}}}}\;{{{{{\rm{specific}}}}}}\;{{{{{\rm{tetramer}}}}}}^{{{{{{\rm{positive}}}}}}\;{{{{{\rm{cells}}}}}}} - {{{{{\rm{\% }}}}}}\;{{{{{\rm{control}}}}}}\;{{{{{\rm{tetramer}}}}}}^{{{{{{\rm{positive}}}}}}\;{{{{{\rm{cells}}}}}}}} \!\right) \times {{{{{\rm{absolute}}}}}}\;{{{{{\rm{number}}}}}}\;{{{{{\rm{of}}}}}}\;{{{{{\rm{CD}}}}}}8^{+} {{{{{{\rm{cells}}}}}}}$. Statistical analysis represents two-way ANOVA with Sidak’s multiple comparison test. Mean and standard deviation are shown. *n* = 37; *p < 0.05; ****p < 0.0001.

Next, the relation between the expansion of adaptive NK cells and HCMV-specific cytotoxic T lymphocytes (HCMV-CTLs) in response to HCMV-R was investigated by tetramer staining (Fig. 2). Whole blood was analyzed for the presence of HCMV-CTLs at the time points indicated in the methods section. The number of tetramers used for each patient was dependent on the HLA typing of both donor and recipient and the commercial availability of HCMV-tetramers for the individual HLA alleles. As shown before (Borchers et al.) the expansion of CMV-CTLs after HCMV-R is an indicator for immunity against recurrent reactivations, therefore the sum of all CD8^+^Tetramer^+^ T cells can be used to assess immunity.

HCMV-CTLs expanded in response to HCMV-R in most patients, while adaptive NK cells expanded to a lesser degree during reactivation. Interestingly, adaptive NK cells expanded significantly when HCMV-R had been resolved and the T cell compartment began to contract (*p* < 0.05).

### Adaptive NK cells degranulate and eliminate HCMV-infected cells

**Fig. 3:**
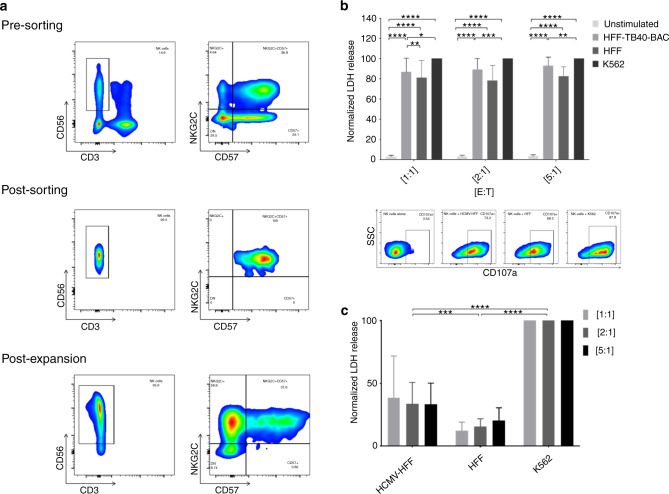
Cytotoxic capacity of adaptive NK cells. In vitro expanded adaptive NK cells were co-cultured with uninfected HFF, HFF-TB40-BAC, or K562 cells at E:T ratios of 1:1; 2:1 and 5:1 as indicated. **a** Representative density plot of NK cells pre- and post-sorting and post-expansion. **b** Normalized percentage of CD107a staining and representative density plot of CD107a surface expression at an E:T ratio of 1:1. **c** The percentage of LDH release was normalized by setting the LDH release in response to K562 to 100%. Statistical analysis represents two-way ANOVA with Tukey’s multiple comparison. Mean and standard deviation are shown. *n* = 7; *p < 0.05; **p < 0.01; ***p < 0.001; ****p < 0.0001.

The notable expansion of adaptive NK cells led to further investigations of their possible role in controlling HCMV-R. Sorted mature adaptive NK cells (NKG2C^+^CD57^+^) were expanded in vitro for 14 days, to obtain sufficient numbers of adaptive NK cells for functional assays. To determine the role of adaptive NK cells in the elimination of HCMV-infected cells, CD107a degranulation assay and the lactate dehydrogenase (LDH) release assay were performed. Figure 3b shows that degranulation of stimulated adaptive NK cells in response to HFF-TB40-BAC was significantly higher than that of adaptive NK cells cultured with uninfected HFF alone at the effector to target ratio of 1:1 (*p* < 0.01). Furthermore, Fig. 3c shows a significant increase of LDH release when adaptive NK cells were co-cultured with HFF-TB40-BAC compared to non-infected HFF (*p* < 0.001) indicating lysis of the target cells.

### Adaptive NK cells recruit T cells in in vitro migration assays

To address the role of adaptive NK cells in HCMV-R and to give a plausible explanation for the marked expansion of absolute cell counts after HCMV-R, we performed migration assays to test the capacity of adaptive NK cells to recruit T cells.

**Fig. 4:**
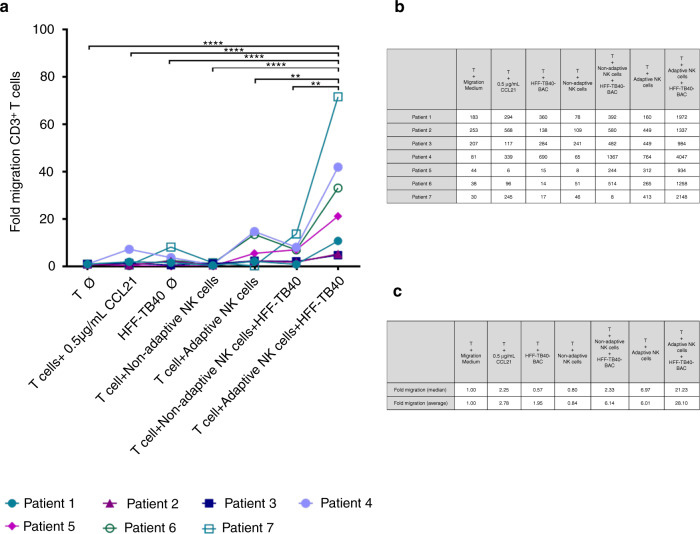
Chemoattractant capacity of adaptive NK cells. **a** The fold migration of T cells in response to medium, the chemokine CCL21 (0.5 µg/ml), HFF-TB40-BAC, non-adaptive NK cells, adaptive NK cells, non-adaptive NK cells and adaptive NK cells co-cultured with HFF-TB40-BAC is shown. **b** Summarized cell counts of migrated T cells. **c** Summarized fold migration (median and average) of T cells. Statistical analysis represents two-way ANOVA with Tukey’s multiple comparison. *n* = 7; **p < 0.01; ****p < 0.0001.

Autologous T cells were cultured in the upper chamber of a transwell system. Cells or chemokines were added to the lower chamber of the transwell system as described in the methods section. The fold migration under the different culture conditions was calculated. Adaptive NK cells cultured without target cells promoted an average 6-fold migration of T cells (Fig. 4c). Co-culture of adaptive NK cells with HFF-TB40-BAC led to a striking increase of 28-fold migration of T cells. This was statistically significant compared to all other conditions tested, and also statistically significantly increased when compared to the migration of the T cells stimulated with the chemoattractant CCL21 used as positive control (*p* < 0.0001).

### Adaptive NK cells secrete a number of activating cytokines/chemokines in transwell assays

**Fig. 5:**
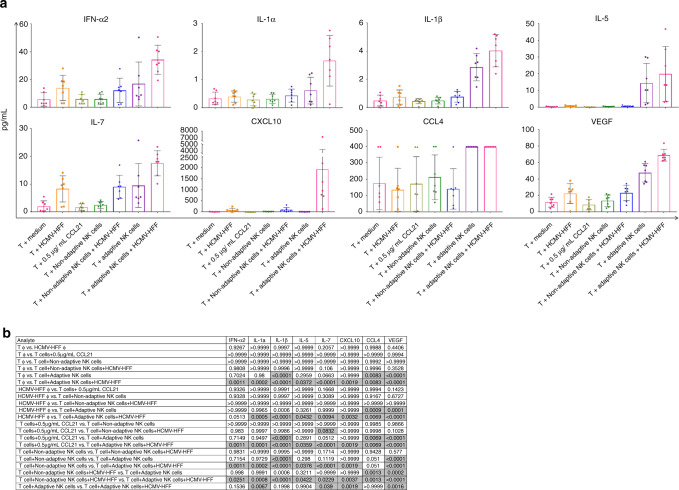
Quantification of cytokines, chemokines and growth factors secreted in transwell assay in pg/ml. **a** Quantification of secreted cytokines and chemokines by T cells with medium; T cells with HFF-TB40-BAC; T cells with 0.5 µg/ml CCL21; T cells with non-adaptive NK cells; T cells with both non-adaptive NK cells and HFF-TB40; T cells with adaptive NK cells and T cells with both adaptive NK cells and HFF-TB40. **b** Summarized *p* values for each of the analytes. Statistical analysis represents one-way ANOVA with Tukey’s multiple comparison. Mean and standard deviations are shown. Gray shaded cells represent statistically significant *p* values; *n* = 7. Below and above detectable levels were replaced by the lowest and highest standard reading, respectively.

To address the excretion of cytokines and chemokines released under the different conditions in the transwell assay the cell-free supernatants were analyzed in a multiplex protein array quantifying 29 cytokines, chemokines and growth factors. Figure 5a shows cytokines and chemokines were excreted at significantly different concentrations when HCMV-stimulated adaptive NK cells were attracting T cells to the lower chambers in vitro.

Eleven of 29 factors analyzed were significantly increased. Five were activating cytokines, such as interferon alpha (IFN-α), IL-1α, IL-1β, IL-7 and the vascular endothelial cell growth factor (VEGF). Those were significantly increased only when adaptive NK cells were co-cultured with HFF-TB40-BAC (Fig. 5a). In addition, adaptive NK cells cultured with target cells led to a significant increase of IL-5 secretion (mean: 19.89 pg/ml, range: 3.11–46.73 pg/ml). The interferon gamma-induced protein 10 (CXCL10) was only secreted at high levels (mean: 1930.16 pg/ml: range: 132.05–7380.23 pg/ml), when adaptive NK cells were co-cultured with HFF-TB40-BAC. Under this condition, T cells had migrated to the bottom wells. T cells cultured with HFF-TB40-BAC showed increased secretion of IFN-α, (mean: 13.88 pg/ml; range: 2.84–287.99 pg/ml), IL-7 (mean: 8.26 pg/ml; range: 1.79–14.09 pg/ml) and VEGF (mean: 22.28 pg/ml; range: 0.82–37.92 pg/ml) too, but at lower levels than in the combination of adaptive NK cells and HFF-TB40-BAC. Interestingly, the secretion of inhibitory cytokines like IL-1 receptor antagonist, IL-10 and IL-12p40 (Supplementary Fig. 1) were increased in the setting of adaptive NK cells stimulated with HFF-TB40-BAC as well. Statistical significance of the different cyto-/chemokines and VEGF is shown in Fig. 5b. Significant *p* values are highlighted in gray.

## Discussion

Here we explored the immune responses of adaptive NK cells triggered by HCMV reactivation.

The current view is that HCMV infection is controlled by HCMV-CTLs [20], but recently adaptive NK cells responsive to HCMV were described [21–23]. Redondo-Pachón et al. and Barron et al., strongly suggest an involvement of adaptive NK cells in the control of HCMV in kidney transplantation and in HSCT, respectively [24, 25]. Our data strongly support the observations of Barron et al. in patients early after HSCT and add to the current literature by including comparisons of immune reconstitution of HCMV-CTL and adaptive NK cells in the immunocompromised host.

The current opinion is that the NK cell compartment consists mainly of immature cells up to 6 months after HSCT [26]. We could show that mature, CD57-positive adaptive NK cells reconstitute early after HSCT in patients with HCMV-R (Fig. 1b). Adaptive NK cell numbers are also increased in healthy HCMV-seropositive individuals compared to seronegative ones, but are stable without HCMV-R [27]. Interestingly, we have shown that adaptive NK cells persist and further expand after resolution of HCMV reactivation in contrast to the retracting HCMV-CTL compartment (Figs. 1a and 2, respectively). This expansion did not affect the absolute cell counts of the total NK cell pool (Fig. 1c), suggesting that antigen/pathogen recognition induces the maturation of adaptive NK cells after HCMV-R (Fig. 1d). This supports Cichocki et al., who observed the expansion of adaptive NK cells after umbilical cord blood transplantation following reduced intensity conditioning [28, 29]. Patients who reactivated HCMV were divided into two groups regarding their early reconstitution of adaptive NK cells. Prior to clinical diagnose of HCMV-R, patients with absolute cell counts of adaptive NK cells equal or above (≥) the average were considered “Responders” and patients below this threshold (<) were considered “Non-Responders”. Only 25% of Responders had multiple HCMV-R, while 52% of Non-Responders developed several HCMV-R. In addition, the duration of HCMV-R in Responders was, on average, ~10 days shorter when compared to Non-Responders (duration of HCMV-R; Non-Responders: 30,075 days (range 6–125); Responders: 20,4 days (range 7–42)). Taken together, these results suggest that the expansion/maturation of adaptive NK cells early on may influence HCMV-immunity, possibly controlling recurrent HCMV-R. HCMV can modulate the host immune response by downregulation of MHC class I molecules (MHC-I) circumventing CD8^+^ T cell recognition [30–32]. This leads in turn to a reduction of NK-inhibitory signals and to NK cell activation. Thus, adaptive NK cells may compensate for the lack of a T cell response due to down regulated MHC-I. On the other hand, HCMV can promote the surface expression of HLA-E thus engaging the NKG2 inhibitory receptor and HCMV expresses a viral homolog that binds to inhibitory receptors with higher affinity than MHC class I molecules [33–36], which could in turn down regulate NK cell responses. The expansion of adaptive NK cells in vivo after HCMV-R argues against this effect in the immunocompromised host (Fig. 1).

In addition, we could show that in vitro expanded, patient-derived adaptive NK cells had an increased cytotoxicity toward HFF-TB40-BAC leading to an increased LDH release (Fig. 3c). This demonstrates the capacity of expanded patient-derived adaptive NK cells to circumvent HCMV evasion mechanisms in vitro. In addition, NK cells are able to recruit other immune cells into the tumor/inflammation microenvironment [37]. To investigate the possible recruitment of T cells by adaptive NK cells in HCMV-infection setting, we performed transwell assays (Fig. 4). We could show that in vitro expanded adaptive NK cells co-cultured with HFF-TB40-BAC were able to promote T cell recruitment to a significantly higher degree when compared to non-adaptive NK cells co-cultured with HFF-TB40-BAC, or adaptive NK cells co-cultured with uninfected HFF. We assume that the cross-talk between the adaptive NK and autologous T cells may have complementary roles in the surveillance of HCMV. Our experiments required target cells for functional assays, which could not be retrieved from the patients. Fibroblast express the MHC class I chain-related protein A and B (MICA/B) on their cell surface, which activate NK cells through binding to NKG2D. We observed an increased expression of NKG2D in the NK subpopulation (data not shown), but we could also show that only the co-cultivation of the expanded adaptive NK cells with HFF-TB40-BAC led to significant increases in cytotoxicity or degranulation, when compared to adaptive NK cells co-cultured with uninfected HFF. One could argue that the virus used to infect HFF may not be comparable to HCMV strains found outside of laboratories. The TB40-BAC-BAC_KL7_-SE-EGFP virus used here contains HCMV genes responsible for the inhibition of both T and NK cell thus is as close to HCMV as currently possible. Therefore, we postulate that the experimental set up had no major influence on the results presented here. It is also noteworthy that the assays can only be performed in vitro and with expanded cells, due to the low frequency of these in immunosuppressed patients up to day +100 after HSCT. We could also show that activating cytokines and chemokines were excreted differentially in the transwell assays (Fig. 5), depending on the presence of adaptive NK cells stimulated with HFF-TB40-BAC. IFN-α as a signature anti-viral cytokine, was secreted by T cells stimulated with HFF-TB40-BAC, as expected [38], but at much lower levels than in the presence of adaptive NK cells. The chemokine CXCL10 directing CXCR3^+^ cells like macrophages, dendritic cells, NK and T cells to inflammation sites [39], was increased significantly only when adaptive NK cells co-cultured with HFF-TB40-BAC attracted T cells to the lower chambers of the transwell assay. CXCL10 is describe to induce apoptosis, to regulate cell growth and proliferation and to promote angiogenesis in infectious and inflammatory diseases [40]. The cytokine excretion pattern of the adaptive NK cells suggests an analogy to Th1 and Th2 cells. Th1 cells produce IFN-γ and other pro-inflammatory cytokines also secreted by the adaptive NK cells when stimulated with HFF-TB40-BAC, while Th2 cells secrete regulatory cytokines like IL-4 and IL-13 to down regulate immune responses. To overcome this regulatory effect Th1-cells excrete CXCL10 [40, 41]. Our data suggests that CXCL10 is produced by stimulated adaptive NK cells, thus possibly recruiting more of Th1 cells to the site of infection. The in vitro expanded adaptive NK cells co-cultured with HFF-TB40-BAC and recruited T cells secreted also increased amounts of inhibitory cytokines like IL1-RA, IL-10 and IL-12p40 (Supplementary Fig. 1). This may suggest a protection mechanism against over-activation and damage prevention by adaptive NK cells, while still maintaining an activating, pro-inflammatory and immune recruitment profile. These results suggest that in vitro expanded adaptive NK cells can recruit T cells due to the increased secretion of several chemo- and cytokines involved in immune modulation. Taken together our results of the in vivo monitoring of adaptive NK cells in patients with and without HCMV-R suggest a role of adaptive NK cells in the immune surveillance of HCMV in immune compromised hosts.

## Patients and methods

### Patient cohort

HCMV-seropositive patients (R+) transplanted between 2016 and 2018 at the Hannover Medical School were eligible for inclusion in this observation study. The study was approved by the Institutional Review Board of the Hannover Medical School (protocol number: 2906). Patients and donors gave written informed consent in accordance with the declaration of Helsinki. Patients and donors were typed for 10 HLA alleles for exon 2 + 3 for HLA-A, B, and C and for exon 2 for HLA-DRB1 and -DQB1 according to the current European Federation for Immunogenetics guidelines. Matched unrelated donors were identical for 10/10 HLA alleles with their recipients. All patients had a follow-up of at least 100 days after HSCT. Patient demographics and transplant procedures are summarized in Table 1. Clinical information on HCMV serostatus of donor (D) and recipient (R), number of patients with HCMV reactivation and day of HCMV reactivation is found on Table 2.

### Monitoring of the NK cell immune reconstitution

PB from HCMV-seropositive patients was collected and analyzed between day 0 and day +100 post-HSCT at intervals of 7–10 days or at the next visit at the out-patient clinic. Erythrocytes were lysed using VersaLyse solution (Beckman Coulter, Marseille, France) and resulting peripheral blood mononuclear cells (PBMCs) were stained for 30 min at room temperature (RT) with the antibodies summarized in Table 3.

**Table 3:** Summarized flow cytometry panel used for the monitoring of adaptive NK cells.

Antibody	Conjugate	Clone	Company
CD3	PerCP	OKT3	Biolegend
CD14	PerCP	HCD14	Biolegend
CD56	APC-Cy7	5.1H11	Biolegend
CD16	PE-Cy7	3G8	Biolegend
CD57	FITC	HCD57	Biolegend
NKG2C	PE	134591	R&D
7AAD	–	–	Biolegend
CD8	AF700	HIT8a	Biolegend
NKG2D	BV421	1D11	Biolegend
NKp46	BV605	9E2	Biolegend

Absolute numbers of adaptive NK cells per µl of whole blood were determined using fluorescent beads (FlowCount Fluorospheres™, Beckman Coulter) and absolute cell number were calculated as previously described [42].

Nine samples (mean: 9; range: 4–12) per patient were analyzed. FACS analysis was performed using FlowJo Version 10 (Treestar, Ashland, USA).

### Monitoring of HCMV infection

PB samples were collected and monitored for immune reconstitution of NK cells and HCMV-CTLs on days 25 (±10), 50 (±10) and 100 (±35) after transplantation, or weekly in case of HCMV reactivation. Erythrocytes were lysed and cells were fixed with VersaLyse (Beckman Coulter) with IOTest®3 fixative solution (Beckman Coulter). Samples were stained for 30 min at RT antibodies and/or tetramers are summarized in Table 4.

**Table 4:** Summarized flow cytometry panel used for the monitoring of HCMV-CTL.

Antibody/HLA-peptide (peptide sequence)	Conjugate	Clone	Company
CD3	PE-Cy7	UCHT1	Beckman Coulter, (Marseille, France)
CD4	PE	13B8.2	Beckman Coulter, (Marseille, France)
CD8	FITC	SFCI21Thy2D3	Beckman Coulter, (Marseille, France)
HLA-A*01:01pp50 (VTEHDTLLY)	PE	3G8	MBL International, (Woburn MA, USA)
HLA-A*02:01 pp65 (NLVPMVATV)	FITC	HCD57	MBL International, (Woburn MA, USA)
HLA-A*02:01 IE1 (VLEETSVML)	PE	134591	MBL International, (Woburn MA, USA)
HLA-A*24:02 pp65 (QYDPVAALF)	PE	–	MBL International, (Woburn MA, USA)
HLA-B*07:02 pp65 (TPRVTGGGAM)	PE	HIT8a	MBL International, (Woburn MA, USA)
HLA-B*08:01 IE1(ELRRKMMYM)	PE	1D11	MBL International, (Woburn MA, USA)
HLA-B*35:01 pp65 (IPSINVHHY)	PE	9E2	MBL International, (Woburn MA, USA)

### Monitoring of HCMV reactivation

PB samples of all patients were monitored weekly for pp65 antigen expression. HCMV reactivation was diagnosed when 5 or more pp65-positive cells per 4 × 10^5^ leukocytes were detected. Reactivation of HCMV was treated with ganciclovir as first line and foscarnet or others as second line therapy.

### Isolation of adaptive NK and T cells

PBMCs from seven patients were isolated by density gradient centrifugation using standard Biocoll (Biochrom, Cambridge, United Kingdom) from PB samples of HCMV-seropositive patients, frozen and stored for analysis. For purification of NK and T cells, PBMCs were thawed and cultured overnight. Next, cells were stained for 10 min at 4 °C with recombinant human antibodies directed against: NKG2C (PE-Vio770 clone: REA205), CD57 (APC-Vio770 clone: REA769), CD3 (VioBlue clone: REA613) and CD56 (PE-Vio615 clone: REA196 all from Miltenyi, Bergisch Gladbach, Germany). Mature adaptive NK cells were sorted as CD3^−^CD56^+^NKG2C^+^CD57^+^; non-adaptive NK cells were sorted as NOT- CD3^−^CD56^+^NKG2C^+^CD57^+^ and T cells were sorted as CD3^+^CD56^−^. Adaptive NK cells were expanded in vitro as described below; non-adaptive NK and T cells cells were cryopreserved for later use.

### Ex vivo expansion of adaptive NK cells

FACS-sorted NKG2C^+^CD57^+^ NK cells were co-cultured with irradiated (25 Gy) allogeneic PBMCs as feeder cells in Miltenyi NK-MACS medium. NK-MACS was supplemented with 1% NK-MACS supplement, 1% Penicillin/Streptomycin (Pen/strep), 5% AB-serum, 500 IU/ml IL-2 (Miltenyi, Bergisch Gladbach, Germany), 10 ng/ml IL-15 (ImmunoTools, Friesoythe, Germany) and 1 µg/ml PHA (Invivogen, Toulouse, France) at 37 °C and 5% CO_2_ in a humidified atmosphere. On day 4, half of the medium was removed and replaced with fresh medium supplemented with1000 IU/ml IL-2 and 20 ng/ml IL-15 and cells were continuously fed every 3 to 4 days. On day 14 of expansion, the phenotype of the cells was assessed by flow cytometry as described above.

### Virus propagation and titration

TB40-BAC-BAC_KL7_-SE-EGFP virus strain used for our experiments was derived from human cytomegalovirus TB40/E was used to establish target cells for functional assays. TB40-BAC-BAC_KL7_-SE-EGFP virus stocks were produced by propagation on human foreskin fibroblast (HFF) as described [43]. HFF were infected at 90–100% confluency with TB40-BAC-BAC_KL7_-SE-EGFP at an MOI of 0.1 and harvested at 100% cytopathic effect. Cellular debris was removed by centrifugation at 2800 × *g* for 10 min. Viral titers were determined by flow cytometric detection of EGFP expressing cells as described [44]. Briefly, 1 × 10^5^ HFF per well were seeded in triplicates into 48-well plates. DMEM supplemented with 10% fetal bovine serum and 1% Pen/Strep (DMEM 10%) was added to the seeded cells together with virus-dilutions ranging from 1×10^0^ to 1×10^−2^ and cells were incubated overnight. Virus inoculum was removed on the next day and cells were cultured for another 24 h in fresh DMEM 10%. Cells were detached with a 0.05% trypsin, 0.02% EDTA solution (Sigma-Aldrich, Taufkirchen, Germany) and EGFP-positive cells were detected and counted by flow cytometry. The concentration of the virus stock was determined as infectious units per milliliter (IU/ml).

### Target cell preparation and NK cell degranulation assay

Confluent HFF were infected at an MOI of 1 for 24 h and the infection rate was assessed by flow cytometry. Cells were thawed and cultured overnight prior to the degranulation assays. Expanded adaptive NK cells were co-cultured with uninfected HFF, HCMV-infected HFF (HFF-TB40-BAC), K562 cells or without target cells as a control at effector: target ratios of 1:1; 2:1 and 5:1. CD107a (PE-Vio770, clone: REA792) was added to the cultures and cells were incubated for 5 h at 37 °C, 5% CO_2_. Cells were washed and stained with antibodies directed against CD3 (Vio-Green, clone: REA613), NKG2C (APC, clone: REA205), CD57 (APC-Vio770, clone: REA769), CD56 (PerCP-Vio700, clone: REA196) and CD8 (VioBlue, clone: REA734) (Miltenyi, Bergisch Gladbach, Germany) for 10 min at 4 °C.

### Lactate dehydrogenase (LDH) assay

In vitro expanded adaptive NK cells were co-cultured with uninfected HFF, HFF-TB40-BAC or K562 cells (positive control) for 5 h, cell-free supernatant was collected and analyzed using the Cytotoxic Detection Kit^Plus^ (LDH) (Roche, Basel, Switzerland) according to the manufacturer’s recommendations. Briefly, 50 µl of reaction mixture was added to 50 µl of cell-free culture supernatant and incubated for 10 min at RT. Stop solution (25 µl) was added and the absorbance was immediately measured at 490 nm in a TECAN Infinite M200 microplate reader (TECAN, Männedorf, Switzerland).

### Transwell assay for in vitro migration of T cells

A transwell assay was used to assess the capacity of in vitro expanded adaptive NK cells to recruit autologous CD3^+^ T cells. Transwell permeable supports 6.5 mm insert, 5.0 µm pore size polycarbonate membranes (Corning, Kaiserslautern, Germany) were used. CD3^+^ T cells were seeded at 5 × 10^5^ cells/ml and added to the top chamber of the transwell. The lower chambers were filled with 500 µl of [1] migration medium, [2] CCL21 (0.5 µg/ml), [3] HFF-TB40-BAC, [4] non-adaptive NK cells, [5] non-adaptive NK cells co-cultured with HFF-TB40-BAC, [6] adaptive NK cells or [7] adaptive NK cells co-cultured with HFF-TB40-BAC. Effector to target ratio for NK cells co-cultured with HFF-TB40-BAC was 2:1 (2.5 × 10^5^ NK cells and 1.25 × 10^5^ target cells). Cells were incubated at 37 °C, 5% CO_2_ for 8 h as described above. The supernatant was collected after 8 h, centrifuged and frozen at −80 °C for further analysis. The bottom chamber was washed twice with PBS and cells were stained with anti-CD3 (PerCP clone: OKT3, BioLegend) and anti-CD8a (Alexa Flour 700 clone: HIT8a, BioLegend). Prior to acquisition, fluorescent counting beads (FlowCount Fluorospheres™, Beckman Coulter) were added to each sample.

### Quantification of cytokines and chemokines

Cyto-/chemokine concentrations in cell-free supernatants were quantified by a multiplex protein array. Milliplex® Map Human Cytokine/Chemokine Magnetic bead panel (Merck Millipore, Darmstadt, Germany) was used according to the manufacturer’s recommendations. Standards were reconstituted in deionized water, diluted from 10.000 pg/ml to 3.2 pg/ml and assay buffer alone was used as background control. Cell-free supernatant (25 µl) was added to U-bottom 96-well plates and mixed with 25 µl of assay buffer. Premixed beads (25 µl) were added to each well and incubated shaking overnight at 4 °C. Plates were washed twice with 200 µl wash buffer. PE-labeled detection antibodies were added to each well and incubated for 1 h at RT. Beads were washed twice and suspended in 150 µl sheath fluid and measured in the Luminex 200 Bioplex reader (Bio Rad, Hercules, CA, United States). Standard curves were calculated according to the five parameter logistic plot.

### Statistical analysis

Statistical analysis was performed using Prism Version 7 (GraphPad Software Inc., California, USA). Tow-way ANOVA and Sidak’s multiple comparison test, two-way ANOVA or one-way ANOVA with Tukey’s multiple comparison test were used. The *p* value <0.05 was considered statistically significant.

## Supplementary information

supplemental Material
