# Supplementary figures and images for: Adaptive NK cells undergo a dynamic modulation in response to human cytomegalovirus and recruit T cells in in vitro migration assays
Source: Bone Marrow Transplant. 2022 Feb 17;57(5):712–20. doi: 10.1038/s41409-022-01603-y (PMC9090630; doi:10.1038/s41409-022-01603-y)

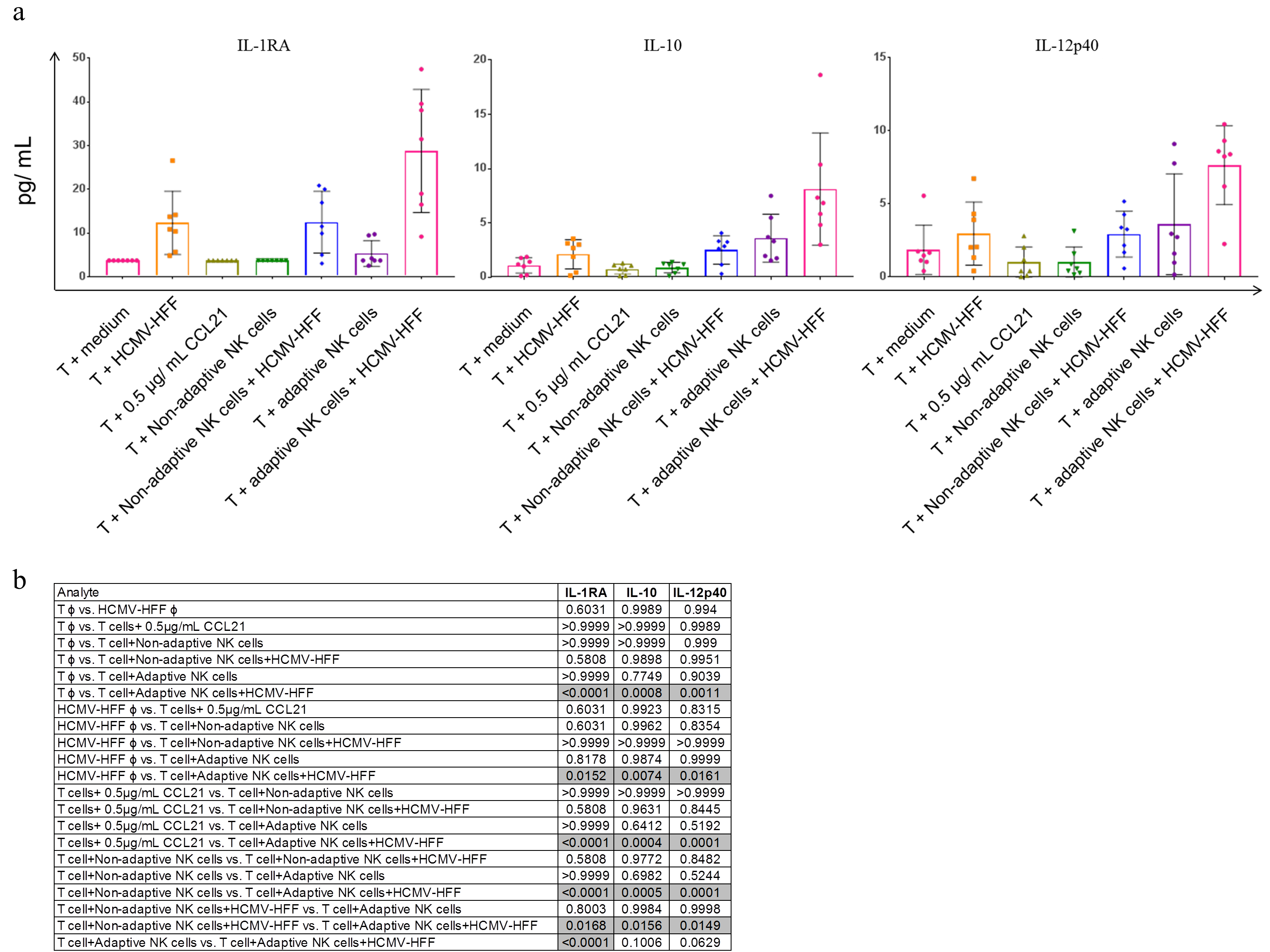

Supplement: Supplementary file 1 — supplemental Material [file 41409_2022_1603_MOESM1_ESM.tif]
